# Supplementary material for: Effect of obstructive sleep apnea on cerebrovascular compliance and cerebral small vessel disease
Source: PLoS One. 2021 Nov 12;16(11):e0259469. doi: 10.1371/journal.pone.0259469 (PMC8589195; doi:10.1371/journal.pone.0259469)
Supplement: S1 Table — (DOCX) [file pone.0259469.s001.docx]

**S1 Table. Correlation coefficients among the continuous or ordinal variables**

|  | MCA PI | *P* | MRIR | *P* | WMH total volume | *P* | WMH subcortical volume | *P* |
| --- | --- | --- | --- | --- | --- | --- | --- | --- |
| Age (year) | 0.480 | <0.001^**^ | 0.031 | 0.765 | 0.430 | <0.001^**^ | 0.512 | <0.001^**^ |
| Total sleep time (min) | -0.217 | 0.033^*^ | 0.029 | 0.779 | -0.223 | 0.028^*^ | -0.224 | 0.027^*^ |
| Sleep efficiency (%) | -0.234 | 0.021^*^ | 0.099 | 0.337 | -0.186 | 0.069 | -0.189 | 0.064 |
| Stage N3 (%) | -0.001 | 0.991 | -0.216 | 0.034^*^ | -0.168 | 0.100 | -0.189 | 0.064 |
| Apnea-hypopnea index (/h) | 0.123 | 0.231 | 0.151 | 0.140 | 0.306 | 0.001^**^ | -0.429 | <0.001^**^ |
| Respiratory distress index (/h) | 0.162 | 0.154 | 0.202 | 0.045^*^ | 0.298 | 0.001^**^ | 0.421 | <0.001^**^ |
| Oxygen desaturation index (/h) | 0.111 | 0.275 | 0.131 | 0.180 | 0.241 | 0.019^*^ | 0.180 | 0.085 |
| Arousal index (/h) | 0.150 | 0.170 | 0.176 | 0.101 | 0.207 | 0.042^*^ | 0.277 | 0.006^*^ |
| MCA MFV (cm/sec) | -0.232 | 0.022^*^ | 0.108 | 0.292 | -0.199 | 0.050 | -0.185 | 0.070 |
| MRIR | 0.138 | 0.177 | – | – | 0.273 | 0.003^**^ | 0.302 | 0.003^**^ |
| MCA PI | – | – | – | – | 0.302 | 0.001^**^ | 0.404 | <0.001^**^ |
|  | WMH periventricular volume | *P* | Total ePVS score | *P* | CS ePVS  score | *P* | BG ePVS score | *P* |
| Age (year) | 0.544 | <0.001^**^ | 0.421 | <0.001^**^ | 0.451 | <0.001^**^ | 0.263 | 0.009^**^ |
| Total sleep time (min) | -0.219 | 0.031^*^ | -0.136 | 0.185 | -0.264 | 0.009^**^ | 0.063 | 0.540 |
| Sleep efficiency (%) | -0.180 | 0.078 | -0.121 | 0.239 | -0.220 | 0.030^*^ | 0.032 | 0.754 |
| Stage N3 (%) | -0.143 | 0.161 | -0.060 | 0.561 | -0.062 | 0.544 | -0.007 | 0.947 |
| Apnea-hypopnea index (/h) | 0.228 | 0.026^*^ | 0.234 | 0.021^*^ | 0.214 | 0.035^*^ | 0.177 | 0.083 |
| Respiratory distress index (/h) | 0.244 | 0.016^*^ | 0.219 | 0.032^*^ | 0.197 | 0.053 | 0.184 | 0.071 |
| Oxygen desaturation index (/h) | 0.251 | 0.013^*^ | 0.198 | 0.058 | 0.176 | 0.093 | 0.189 | 0.065 |
| Arousal index (/h) | 0.128 | 0.213 | 0.133 | 0.195 | 0.172 | 0.092 | 0.039 | 0.708 |
| MCA MFV (cm/sec) | -0.213 | 0.037^*^ | -0.130 | 0.205 | -0.161 | 0.116 | -0.049 | 0.635 |
| MRIR | 0.301 | 0.003^**^ | 0.377 | <0.001^**^ | 0.270 | 0.008^**^ | 0.434 | <0.001^**^ |
| MCA PI | 0.416 | <0.001^**^ | 0.412 | <0.001^**^ | 0.432 | <0.001^**^ | 0.267 | 0.008^**^ |

MCA: middle cerebral artery, PI: pulsatility index, MRIR: mean middle cerebral artery resistance index ratio, WMH: white matter hyperintensity, ePVS: enlarged perivascular space, CS: centrum semiovale, BG: basal ganglia, and MFV: mean flow velocity. ^*^*P*<0.05 and ^**^*P*<0.01.
