# Supplementary material for: Effect of obstructive sleep apnea on cerebrovascular compliance and cerebral small vessel disease
Source: PLoS One. 2021 Nov 12;16(11):e0259469. doi: 10.1371/journal.pone.0259469 (PMC8589195; doi:10.1371/journal.pone.0259469)
Supplement: S2 Table — (DOCX) [file pone.0259469.s002.docx]

**S2 Table. Univariate analyses for categorical parameters associated with white-matter hyperintensity volume**

|  | MCA PI | | | MRIR | | | WMH total volume (%) | | | WMH subcortical volume (%) | | |
| --- | --- | --- | --- | --- | --- | --- | --- | --- | --- | --- | --- | --- |
|  | Yes | No | *P* | Yes | No | *P* | Yes | No | *P* | Yes | No | *P* |
| Male sex | 0.80±0.15 | 0.80±0.13 | 0.928 | 1.00±0.04 | 0.98±0.06 | 0.111 | 0.47±0.41 | 0.54±0.42 | 0.435 | 0.26±0.22 | 0.28±0.22 | 0.656 |
| Hypertension | 0.82±0.16 | 0.78±0.11 | 0.226 | 0.99±0.05 | 1.00±0.04 | 0.145 | 0.51±0.45 | 0.48±0.35 | 0.721 | 0.27±0.24 | 0.25±0.18 | 0.580 |
| Diabetes mellitus | 0.83±0.14 | 0.79±0.15 | 0.173 | 0.99±0.04 | 0.99±0.05 | 0.830 | 0.68±0.56 | 0.42±0.30 | 0.025^*^ | 0.36±0.3 | 0.22±0.15 | 0.025^*^ |
| Hyperlipidemia | 0.82±0.14 | 0.80±0.15 | 0.398 | 0.99±0.04 | 0.99±0.05 | 0.916 | 0.69±0.58 | 0.42±0.29 | 0.022^*^ | 0.29±0.12 | 0.26±0.22 | 0.887 |
| Heart failure | 0.81±0.23 | 0.80±0.14 | 0.983 | 1.00±0.04 | 0.99±0.05 | 0.629 | 1.05±0.88 | 0.48±0.39 | 0.378 | 0.54±0.45 | 0.26±0.21 | 0.392 |
| Atrial fibrillation | 0.86±0.26 | 0.80±0.13 | 0.594 | 1.04±0.04 | 0.99±0.05 | 0.012 | 0.46±0.16 | 0.50±0.43 | 0.796 | 0.24±0.08 | 0.27±0.23 | 0.768 |
|  | WMH periventricular volume (%) | | | Total ePVS score | | | CS ePVS score | | | BG ePVS score | | |
|  | Yes | No | *P* | Yes | No | *P* | Yes | No | *P* | Yes | No | *P* |
| Male sex | 0.22±0.19 | 0.26±0.20 | 0.254 | 4 [2–5] | 4 [2–5] | 0.774 | 2 [1–3] | 2 [1–3] | 0.994 | 1 [1–2] | 1 [1–2] | 0.511 |
| Hypertension | 0.24±0.21 | 0.23±0.17 | 0.892 | 3 [2–5] | 4 [2–4.5] | 0.288 | 2 [1–3] | 2 [1–3] | 0.986 | 1 [1–2] | 2 [1–2] | 0.017^*^ |
| Diabetes mellitus | 0.32±0.26 | 0.2±0.15 | 0.027^*^ | 4 [3–5] | 3.5 [2–4] | 0.040^*^ | 3 [2–3] | 2 [1–3] | 0.018^*^ | 1 [1–2] | 1 [1–2] | 0.304 |
| Hyperlipidemia | 0.19±0.11 | 0.24±0.20 | 0.815 | 4 [3–5] | 4 [2–5] | 0.735 | 3 [2–3] | 2 [1–3] | 0.382 | 1 [1–2] | 1 [1–2] | 0.576 |
| Heart failure | 0.51±0.43 | 0.23±0.18 | 0.364 | 4 [0–4] | 4 [2–5] | 0.896 | 3 [0–3] | 2 [1–3] | 0.934 | 1 [0–1] | 1 [1–2] | 0.867 |
| Atrial fibrillation | 0.22±0.08 | 0.24±0.2 | 0.830 | 4 [3–5] | 4 [2–5] | 0.241 | 2 [2–3] | 2 [1–3] | 0.342 | 2 [1–2] | 1 [1–2] | 0.233 |

Data are reported as mean (standard deviation). MCA: middle cerebral artery, PI: pulsatility index, MRIR: mean middle cerebral artery resistance index ratio, WMH: white matter hyperintensity, ePVS: enlarged perivascular space, CS: centrum semiovale, and BG: basal ganglia. ^*^*P*<0.05.
