# Supplementary material for: Effect of obstructive sleep apnea on cerebrovascular compliance and cerebral small vessel disease
Source: PLoS One. 2021 Nov 12;16(11):e0259469. doi: 10.1371/journal.pone.0259469 (PMC8589195; doi:10.1371/journal.pone.0259469)
Supplement: S3 Table — (DOCX) [file pone.0259469.s003.docx]

**S3 Table. Profiles of clinical, polysomnography, and transcranial cerebrovascular compliance markers in patients with or without cerebral microbleeds or lacunes**

|  | CMB (+)  (N=9) | CMB (-) (N=88) | *P* | Lacune (+)  (N=11) | Lacune (-)  (N=86) | *P* |
| --- | --- | --- | --- | --- | --- | --- |
| Age (years) | 64.1±10.9 | 64.8±11.1 | 0.858 | 62.5±9.3 | 65±11.2 | 0.485 |
| Male sex (%) | 6 (66.7) | 53 (60.2) | 0.710 | 8 (72.7) | 51 (59.3) | 0.396 |
| Hypertension (%) | 6 (66.7) | 54 (61.4) | 0.758 | 6 (54.5) | 54 (62.8) | 0.601 |
| Diabetes mellitus (%) | 3 (33.3) | 26 (29.5) | 0.815 | 4 (36.4) | 25 (29.1) | 0.623 |
| Hyperlipidemia (%) | 2 (22.2) | 27 (30.7) | 0.751 | 3 (27.2) | 26 (30.2) | 0.723 |
| Heart failure (%) | 0 (0.0) | 3 (3.4) | 0.578 | 1 (9.1) | 2 (2.3) | 0.227 |
| Atrial fibrillation (%) | 0 (0.0) | 7 (8.0) | 0.385 | 1 (9.1) | 6 (7.0) | 0.801 |
| Time in bed (min) | 422.2±26.2 | 420.1±46.5 | 0.897 | 425.3±43.0 | 419.7±45.4 | 0.701 |
| Total sleep time (min) | 303.4±59.0 | 324.5±64.6 | 0.349 | 310.4±55.9 | 324.1±65.2 | 0.508 |
| Sleep efficiency (%) | 72.1±14.5 | 77.7±13.9 | 0.255 | 73.4±14.2 | 77.7±14.0 | 0.340 |
| Stage N1 (%) | 23.1±18.5 | 21.2±13.0 | 0.699 | 25.5±17.2 | 20.9±13.0 | 0.288 |
| Stage N2 (%) | 43.1±11.3 | 53.9±63.0 | 0.631 | 45.3±12.7 | 54.0±64.1 | 0.653 |
| Stage N3 (%) | 18.0±11.5 | 15.0±9.4 | 0.378 | 15.7±5.8 | 15.3±10.0 | 0.892 |
| Stage REM sleep (%) | 16.6±8.5 | 19.0±8.5 | 0.415 | 13.5±9.3 | 19.4±8.2 | 0.068 |
| Sleep-onset latency (min) | 13.8±16.4 | 12.0±12.6 | 0.683 | 7.5±11.9 | 12.7±13.0 | 0.210 |
| REM sleep latency (min) | 102.3±66.5 | 110.3±73.3 | 0.756 | 110.1±85.9 | 109.5±71.1 | 0.977 |
| Apnea-hypopnea index (/h) | 21.8±22.5 | 18.8±17.9 | 0.639 | 25.0±22.7 | 18.3±17.6 | 0.256 |
| Respiratory distress index (/h) | 22.0±2.2 | 19.8±17.9 | 0.723 | 25.8±23.4 | 19.2±17.5 | 0.260 |
| Oxygen desaturation index (/h) | 24.0±23.0 | 19.6±18.0 | 0.435 | 27.6±23.0 | 19.0±18.0 | 0.223 |
| Arousal index (/h) | 24.5±15.6 | 25.1±15.0 | 0.920 | 29.0±18.7 | 24.5±14.5 | 0.348 |
| MCA MFV (cm/sec) | 50.7±11.7 | 52.5±17.1 | 0.756 | 48.5±14.3 | 52.8±16.9 | 0.421 |
| MCA PI | 0.86±0.15 | 0.80±0.14 | 0.246 | 0.82±0.10 | 0.80±0.15 | 0.630 |
| MRIR | 0.97±0.05 | 0.99±0.05 | 0.275 | 0.99±0.02 | 0.99±0.05 | 0.689 |

Data are reported as number (percentage) or as mean± standard deviation. CMB: cerebral microbleeds, REM: rapid eye movement, MCA: middle cerebral artery, MFV: mean flow velocity, PI: pulsatility index, and MRIR: mean middle cerebral artery resistance index ratio.
