# Supplementary material for: Effect of obstructive sleep apnea on cerebrovascular compliance and cerebral small vessel disease
Source: PLoS One. 2021 Nov 12;16(11):e0259469. doi: 10.1371/journal.pone.0259469 (PMC8589195; doi:10.1371/journal.pone.0259469)
Supplement: Attachment — Submitted filename: PONE-D-21-27488-Response to Reviewer Comments.docx [file pone.0259469.s006.docx]

**Effect of obstructive sleep apnea on cerebrovascular compliance and cerebral small vessel disease**

**Manuscript No.: PONE-D-21-27488**

We are grateful for the thorough review and kind advices that were of great help to improve the quality of our manuscript. The manuscript was revised to clarify the issues raised by the reviewers. The changes and authors’ opinions according to the reviewers’ comments are summarized below. We also enabled the "track changes" feature in the manuscript.

**REVIEWER #1:**
General Comments:

Interesting paper, minor corrections to improve the quality

Specific Comments:
#1. Introduction
-Line 55, Subcortical ischemic vascular dementia (SIVD) is very common among older people, but has no approved treatment. A recent randomized study has shown that remote ischemic conditioning (RIC) reduces the recurrence of ischemic stroke and is an effective therapy for patients with SIVD. please cite doi: 10.1186/s12883-019-1435-y.

Line 60, elderly patients are known to have an increased risk of OSAS, with a prevalence of more severe forms after 65 than younger patients. The same severity of obstructive sleep apnea has also been shown to be related to olfactory disorders in the patient with obstructive sleep apnea, the origin of which could be attributed to disorders of the central nervous system. please cite doi:10.1007/s00405-020-06316-w.

Line 70, please cite doi:10.14639/0392-100X-895

**Response: We thank the reviewer for this discerning advices and recommendation of important up-to-date articles regarding the pathomechanism of OSA. We revised the relevant sentences in the **Introduction** and added the articles the reviewer has recommended.

#2. Line 311, also surgical approaches demonstrated improvement of obstructive sleep apnea syndrome (OSAS) and changes in high sensitivity C-reactive protein (hs-CRP) concentrations have been reported after transfer pharyngoplasty (RP), an elevated variant of uvulopalatopharyngoplasty, reducing cardiovascular risk in subjects with obstructive sleep apnea.
please cite doi:10.1177/0194599810395104 and doi:10.1007/s00405-020-05883-2

**Response: We again thank the reviewer for this comment. We revised the **Discussion** section and added the articles the reviewer has recommended.

**REVIEWER #2:**
General Comments:
The paper examined an interesting topic. I support its approval suggesting some minor corrections to improve the quality.

Specific Comments:
1. Introduction:
Line 60: Reported incidence of OSA is from 5.6% to 60% in people over 65 (cite doi: 10.3390/ijerph17031120) while SVD presents an increased one from 6% at age 60 to 28% at age 80 (cite doi: 10.3390/ijerph17031120).
Line 70: The endothelial dysfunction, may be related to the consequent generation of reactive oxygen species (ROS) and pro-inflammatory molecules, resulting in microvascular damage in OSA patients (cite doi.org/10.3390/jcm10020277).
**Response: We thank the reviewer for this comments and recommendation of important up-to-date articles regarding the pathomechanism of OSA. We revised the relevant sentences in the **Introduction** and added the articles the reviewer has recommended.

2. Line 295: A recent meta-analysis reported that moderate to severe OSA is positively associated with WMH and SBI but not CMBs or PVS (cite doi: 10.1093/sleep/zsz264).

**Response: We thank the reviewer for informing us about this article. According to the reviewer’s recommendation, we revised the relevant sentences and added the articles the reviewer has recommended.

3. In all paper put the dots after the brackets.

**Response: We applied the changes the reviewer has requested, throughout the manuscript.

Once again, we thank the editor and the reviewers for the thoughtful comments about our work. We hope that this revision will meet the reviewers' approval.

Sincerely,

Yong-Seok Lee, MD, PhD

Department of Neurology, College of Medicine Seoul National University, Seoul National University-Seoul Metropolitan Government Boramae Medical Center

Dongjak-gu, Shindaebang-dong, Boramae 5 Road 20, 156-707, Seoul, Republic of Korea

Tel: 82-2-870-2476, Fax: 82-2-831-2826, E-mail: mercades@snu.ac.kr

And

Hyun-Woo Nam, MD, PhD

Department of Neurology, College of Medicine Seoul National University, Seoul National University-Seoul Metropolitan Government Boramae Medical Center

Dongjak-gu, Shindaebang-dong, Boramae 5 Road 20, 156-707, Seoul, Republic of Korea

Tel: 82-2-870-2471, Fax: 82-2-831-2826, E-mail: hwnam85@gmail.com
